# Incidence of Median and Ulnar Neuropathy Following Nonupper Extremity Surgery

**DOI:** 10.1016/j.jhsg.2026.100972

**Published:** 2026-02-27

**Authors:** Eric J. Gullborg, Kyleen Jan, Jason H. Kim, Christopher B. Song, Robert W. Wysocki, John J. Fernandez, Mark S. Cohen, Xavier C. Simcock

**Affiliations:** Department of Orthopaedic Surgery, Rush University Medical Center, Chicago, IL

**Keywords:** Carpal tunnel syndrome, Cubital tunnel syndrome, Peripheral neuropathy, Postoperative complications, Upper-extremity neuropathy

## Abstract

**Purpose:**

Carpal tunnel syndrome (CTS) and cubital syndrome (CuTS) are common neuropathies typically associated with upper-extremity surgeries or systemic risk factors. This study investigates the incidence of CTS and CuTS following anterior cervical discectomy and fusion (ACDF), total hip arthroplasty (THA), and total knee arthroplasty (TKA) to evaluate whether postoperative upper-extremity neuropathy is linked to perioperative systemic responses, patient-specific risk factors, or a combination of the above.

**Methods:**

Patients who underwent ACDF, primary THA, or primary TKA at a single institution between 2015 and 2025 were retrospectively reviewed. Cases of postoperative CTS or CuTS were identified based on clinical presentation and electrodiagnostic testing when available. Each chart was reviewed to confirm the diagnosis, timing, and extract clinical details. Demographic, diagnostic, surgical, and clinical outcomes were compared between cohorts.

**Results:**

Among 49,752 patients, 463 (0.93%) developed postoperative CTS and/or CuTS. The observed incidence was highest in the ACDF cohort (3.05%) compared to TKA (0.88%) and THA (0.82%). CTS accounted for 78% of cases, CuTS for 11%, and combined CTS/CuTS for 11%. Neuropathy distribution varied considerably by surgical group, with ACDF patients having higher rates of CuTS and combined CTS/CuTS diagnoses. Time to diagnosis was shorter in the ACDF group compared to other groups. Surgical decompression was performed in 69.5% of cases, with symptom resolution in 90.4%. Of the 30.5% managed conservatively, 34.8% reported persistent symptoms.

**Conclusions:**

Upper-extremity neuropathy can develop following nonupper extremity surgeries, particularly ACDF. The timing and pattern suggest that perioperative systemic and patient-level factors may contribute more substantially toward neuropathy development than local surgical effects.

**Type of study/level of evidence:**

Prognostic III.

Carpal tunnel syndrome (CTS) and cubital tunnel syndrome (CuTS) are the most common entrapment neuropathies of the upper extremity, with CTS being the most common worldwide.^1^ The prevalence of CTS in the general adult population is estimated to be between 2.7% and 4.9%, whereas the annual incidence of CuTS is approximately 30 cases per 100,000 individuals.^1^^,^^2^ CTS is characterized by compression of the median nerve at the level of the volar wrist within the carpal tunnel, whereas CuTS involves ulnar nerve compression at the medial elbow within the cubital tunnel. Both conditions have been reported to be associated with various risk factors such as repetitive use, advanced age, obesity, diabetes, pregnancy, and prior upper-extremity surgery.^3^^,^^4^

The development of CTS and CuTS following upper-extremity procedures has been frequently studied. One study reported a 0.76% incidence of distal peripheral neuropathy among nearly 10,000 patients undergoing arthroscopic shoulder surgery.^5^ Another study found that the incidence of CTS was considerably higher in the ipsilateral limb within 1 year of arthroscopic rotator cuff or labral repair, compared to the nonsurgical contralateral side.^4^ Hypothesized mechanisms include intraoperative malpositioning or dependent edema, but there is little evidence to elucidate how much those factors play a role compared to other possible factors, such as a systemic inflammatory response. Overall, the existing literature on distal upper-extremity neuropathies after common nonupper extremity orthopedic procedures is lacking.^5^

As such, the primary objective of this study was to (1) describe the observed incidence of CTS and CuTS following nonupper extremity surgeries such as anterior cervical discectomy and fusion (ACDF), total hip arthroplasty (THA), or total knee arthroplasty (TKA) and (2) assess patient response to decompression surgery. These procedures were selected because they represent high-volume elective surgeries, span distinct anatomical regions, and involve different perioperative positioning, operative duration, and physiological stressors. We hypothesized that CTS and CuTS would occur at a rate similar to that after upper-extremity surgery as available in the literature and that surgical decompression would be widely successful in symptom resolution.

## Materials and Methods

### Patient selection

Postoperative peripheral nerve compression was defined as new onset numbness, paresthesia, pain, or motor weakness in the median or ulnar nerve distribution after the index surgery, confirmed clinically by the treating physician and, when available, electrodiagnostic testing. A retrospective review was performed of patients who underwent ACDF, THA, or TKA at a single institution from 2015 to 2025 using Current Procedural Terminology codes 22551, 22630, 27130, 27445, and 27447. These results were then followed by another query for International Classification of Diseases, 10th Revision (ICD10) diagnosis codes of carpal tunnel (G56.00, G56.01, G56.02); median nerve (G56.10, G56.11, G56.12); and ulnar nerve (G56.20, G56.21, G56.22). All new diagnosis of CTS or CuTS documented after the index procedure through January 2025 were included.

Diagnoses of CTS and CuTS were made clinically by the treating physician and were retrospectively confirmed though detailed chart review, requiring documentation of findings consistent with median and ulnar nerve compression. Electrodiagnostic testing was used to support the diagnosis when available. For each patient identified through Current Procedural Terminology and International Classification of Diseases, 10th Revision queries, the full clinical record was examined to verify the timing of symptoms relative to index surgery and confirm that neuropathic symptoms were new. The date of formal diagnosis was used as a standardized proxy for symptom onset, as exact symptom onset dates were inconsistently documented. Patients with any documentation of preoperative numbness, paresthesia, weakness, prior evaluation or treatment for compressive neuropathy in these distributions, or preoperative electrodiagnostic studying were excluded. For patients with multiple qualifying index surgeries (eg, both THA and TKA), the most recent surgery prior to diagnosis of peripheral neuropathy was used for analysis. Patients who underwent nonqualifying surgical procedures (eg, rotator cuff repair) between the index surgery and the diagnosis of peripheral neuropathy were excluded. Institutional Review Board approval was obtained.

### Study variables

Patient demographics were recorded including age at neuropathy diagnosis, biological sex, race, and body mass index (BMI). The type of index surgery (ACDF, THA, or TKA) was documented for all patients. Peripheral neuropathy characteristics were classified by type (CTS, CuTS, or both), laterality (unilateral or bilateral), and time from surgery to neuropathy diagnosis (months). When available, EMG results were categorized as normal-to-mild or moderate-to-severe based on physician interpretation. Treatment modality after diagnosis (conservative vs surgical decompression) and resolution of symptoms were recorded. Conservative treatment included splinting, oral or injected corticosteroids, and clinical observation and follow-up. Symptom resolution was defined as complete or near complete improvement in sensory and/or motor findings documented in follow-up notes or, when available, by improvement in EMG results.

### Statistical analysis

Demographic and diagnostic data were collected and anonymized. Comparisons across surgical cohorts and neuropathy subtypes were performed as secondary descriptive analyses using chi-square or Fisher exact tests for categorical variables and *t* tests, analysis of variance, or Kruskal–Wallis for continuous variables. Post hoc pairwise comparisons were performed where appropriate. A *P* value < .05 was considered statistically significant.

## Results

In total, 1,638 ACDF, 17,008 THA, and 31,106 TKA procedures were performed from 2015 to 2025 (49,752 total). Of these, 463 patients met inclusion criteria after query and review for CTS and/or CuTS: 50 after ACDF, 139 after THA, and 247 after TKA.

### Demographics

Mean age at diagnosis was 65.7 ± 9.2 years. ACDF patients were younger (60.9 ± 10.2) than THA (66.4 ± 10.6, *P* = .002) and TKA (66.5 ± 8.3 years, *P* = .004) patients. Sex distribution was similar (47.7% men and 52.3% women). Mean BMI was 32.5 ± 6.7 kg/m^2^ and differed by cohort: TKA (33.6 ± 6.5 kg/m^2^) had the highest BMI compared with THA (31.3 ± 6.9 kg/m^2^, *P* = .002) and ACDF (30.0 ± 7.4 kg/m^2^, *P* = .001) (Table 1).

**Table 1:** Demographic Data

Characteristic	Total (n = 463)	ACDF (n = 50)	THA (n = 139)	TKA (n = 247)	*P* Value
Age∗	65.7 ± 9.2	60.9 ± 10.2	66.4 ± 10.6	66.5 ± 8.3	.002†
Sex^‡^					
Male	47.7% (221)	50% (25)	43.2% (60)	49.6% (136)	.44
Female	52.3% (242)	50% (25)	56.8% (79)	50.4% (138)	
Race‡					
White	83.4% (386)	88.0% (44)	82.0% (114)	83.2% (228)	.35
Black	11.9% (55)	6.0% (3)	15.1% (21)	11.3% (31)	
Other	4.7% (22)	6.0% (3)	2.9% (4)	5.5% (15)	
BMI∗	32.5 ± 6.7	30.0 ± 7.4	31.3 ± 6.9	33.6 ± 6.5	<.001†

ACDF, anterior cervical discectomy and fusion; THA, total hip arthroplasty.

∗ Values are given as mean ± standard deviation.

† *P* value < .05 indicates statistical significance.

‡ Values are given as percentages with the number in parentheses.

### Incident rates and neuropathy

Overall distal peripheral neuropathy incidence was 0.93% (463/49,752) and varied across surgical cohorts, with a higher observed incidence after ACDF (3.05%) compared with THA (0.82%) and TKA (0.88%). Mean time from surgery to diagnosis was 27.3 ± 22.6 months. ACDF patients were diagnosed sooner than TKA patients (18.6 ± 18.2 vs 29.4 ± 23.2 months, *P* = .002). Bilateral involvement occurred in 35.9% of patients. Neuropathy distribution differed across cohorts: ACDF patients had more isolated CuTS than TKA (*P* = .02) and more combined CTS/CuTS diagnoses than both THA (*P* = .01) and TKA patients (*P* = .04). THA patients showed more isolated CuTS than TKA patients (*P* = .04), whereas combined diagnoses were more frequent in TKA than THA (*P* = 0.04; Table 2).

**Table 2:** Neuropathy Incidence Rates and Characteristics

Characteristic	Total (n= 463)	ACDF (n = 50)	THA (n = 139)	TKA (n = 247)	*P* Value
Rate of DPN∗	0.93% (463/49,752)	3.05% (50/1,638)	0.82% (139/17,008)	0.88% (274/31,106)	
Time from surgery to diagnosis (mo)†	27.3 ± 22.6	18.6 ± 18.2	26.2 ± 23.0	29.4 ± 23.2	.002‡
Type of DPN∗					
CTS	78.4% (363/463)	62.0% (31/50)	77.7% (108/139)	81.8% (224/274)	.005‡
CuTS	11.0% (51/463)	18.0% (9/50)	15.1% (21/139)	7.7% (21/274)	
Both	10.6% (49/463)	20.0% (10/50)	7.2% (10/139)	10.6% (29/274)	
Laterality∗					
Unilateral	64.1% (297/463)	66.0% (33/50)	66.9% (93/139)	62.4% (171/274)	.48
Bilateral	35.9% (166/463)	34.0% (17/50)	33.1% (46/139)	37.6% (103/274)	

ACDF, anterior cervical discectomy and fusion; Both, carpal tunnel and cubital tunnel syndrome; DPN, distal peripheral neuropathy; THA, total hip arthroplasty.

∗ Values are given as percentages with the proportion in parentheses.

† Values are given as mean ± standard deviation.

‡ *P* value < .05 indicates statistical significance.

### Decompression rate

Decompression rates varied by neuropathy type. Patients with CTS (71.3%) and combined CTS/CuTS (73.5%) underwent surgery more often than patients with isolated CuTS (52.9%; *P* = .009, *P* = .04). Decompression rates were similar among surgical cohorts (ACDF 68.0%, THA 67.6%, and TKA 71.1%). Patients with moderate-to-severe EMG findings underwent decompression more frequently (82.0%) than those with normal-to-mild EMG results (49.2%; *P* < .001; Table 3).

**Table 3:** Surgical Decompression Rate

Neuropathy type	CTS	CuTS	Both	*P* value
Decompression	71.3% (259/363)	52.9% (27/51)	73.5% (36/49)	.03∗†
				
Index surgery	ACDF	THA	TKA	*P* value
Decompression	68% (34/50)	67.6% (94/139)	71.1% (194/274)	.77
				
EMG severity	EMG normal/mild	EMG moderate/severe		*P* value
Decompression	49.2% (30/61)	82.0% (201/245)		<.001∗

ACDF, anterior cervical discectomy and fusion; Both, carpal tunnel and cubital tunnel syndrome; THA, total hip arthroplasty.

Note: Values are given as percentages with the proportion in parentheses.

∗ *P* value < .05 indicates statistical significance.

† *P* value was 0.009 when comparing CTS and CuTS, 0.04 when comparing CuTS and Both.

### Symptom resolution

In total, 52 patients were lost to follow-up. Of the remainder, 30.5% were treated conservatively and 69.5% surgically. In conservatively treated patients, resolution occurred in 31.7% of CTS, 26.1% of CuTS, and 21.4% of combined cases, with similar rates across surgical cohorts. EMG data were available for 75 conservatively treated cases; resolution was more frequent with normal-to-mild findings (45.2%) than with moderate-to-severe changes (27.3%; Table 4).

**Table 4:** Rate of Symptom Resolution With Conservative Versus Surgical Management

Conservative Management				
	CTS	CuTS	Both	*P* Value
Symptom resolution	31.7% (33/104)	26.1% (6/23)	21.4% (3/14)	.75
	ACDF	THA	TKA	*P* Value
Symptom resolution	25.0% (4/16)	33.33% (15/45)	30.0% (24/80)	.84
	EMG normal/mild	EMG moderate/severe		*P* Value
Symptom resolution	45.2% (14/31)	27.3% (12/44)		.15
Surgical Management				
	CTS	CuTS	Both	*P* Value
Symptom resolution	89.6% (232/259)	85.2% (23/27)	88.9% (32/36)	.67
	ACDF	THA	TKA	*P* Value
Symptom resolution	85.3% (29/34)	87.2% (82/94)	92.3% (179/194)	.2
	EMG normal/mild	EMG moderate/severe		*P* Value
Symptom resolution	96.7% (29/30)	88.6% (178/201)		.33

ACDF, anterior cervical discectomy and fusion; Both, carpal tunnel and cubital tunnel syndrome; THA, total hip arthroplasty.

Note: Values are given as percentages with the proportion in parentheses.

Surgical decompression achieved higher rates of symptom resolution (*P* < .001). Improvement occurred in 89.6% of CTS, 85.2% of CuTS, and 88.9% of combined cases with comparable resolution across surgical subgroups. Among 231 surgical patients with EMG data, 96.7% of those with normal-to-mild findings and 88.6% with moderate-to-severe findings achieved symptom resolution (Table 4).

### EMG severity

EMG data were available for 306 patients (66.1%): 61 normal-to-mild, 245 moderate-to-severe. Patients with moderate-to-severe findings were older (66.68 ± 9.08 vs 62.56 ± 11.13 years, *P* = .017). Severity was unrelated to index surgery type but differed by neuropathy pattern (*P* = .0002). Isolated CTS (81.2%, *P* < .001) and combined CTS/CuTS (14.7%, *P* < .001) were more often moderate-to-severe on EMG than isolated CuTS (4.1%; Table 5).

**Table 5:** EMG Severity and Characteristics

Characteristic	EMG Normal/Mild	EMG Moderate/Severe	*P* Value
Age∗	62.56 ± 11.13	66.68 ± 9.08	.017†
Type of surgery‡			
ACDF	28.9% (11/38)	71.1% (27/38)	.25
THA	20.9% (19/91)	79.1% (72/91)	
TKA	17.5% (31/177)	82.5% (146/177)	
Type of DPN‡			
CTS	68.9% (42/61)	81.2% (199/245)	<.001†
CuTS	21.3% (13/61)	4.1% (10/245)	
Both	9.8% (6/61)	14.7% (36/245)	

ACDF, anterior cervical discectomy and fusion; Both, carpal tunnel and cubital tunnel syndrome; DPN, distal peripheral neuropathy; THA, total hip arthroplasty.

∗ Values are given as mean ± standard deviation.

† P value < .05 indicates statistical significance.

‡ Values are given as percentages with the proportion in parentheses.

## Discussion

It is commonly postulated that local inflammation or dependent edema after upper-extremity surgery can contribute to the development of CTS or CuTS. However, their occurrence following surgeries unrelated to the upper extremity highlights a more complex interplay of systemic, positional, and patient-specific variables. In this study, we explored the incidence and characteristics of distal upper-extremity neuropathies following ACDF, THA, and TKA, aiming to describe the potential influence of surgical type and perioperative conditions on the emergence and progression of these nerve compression syndromes.

We found that although these neuropathies do occur following nonupper extremity surgery, they are uncommon, with an overall incidence of 0.93% occurring at a mean 27.3 ± 22.6 months after surgery. Importantly, this incidence is comparable to CTS or CuTS development after upper-extremity surgery seen in several other studies.^4^^,^^5^^,^^6^, ^7^, ^8^ This may suggest that surgery itself, whether involving the upper or lower extremity, is unlikely to be a major independent risk factor for the development of CTS or CuTS. In a retrospective review of over 9,800 patients from a multi-surgeon database, Dua et al^5^ found that the incidence of compression peripheral neuropathy after shoulder surgery was 0.76%. They found that 88.6% of patients who underwent surgical decompression had complete symptom resolution, which is consistent with our findings.^5^ The authors proposed several explanations, including downstream hand swelling or tight wrapping of the upper extremity in a robotic arm positioner. However, the similar incidence observed across disparate surgical procedures in our cohort, together with the prolonged interval between index surgery and neuropathy diagnosis, supports the interpretation that postoperative CTS and CuTS most often reflect baseline susceptibility rather than procedure-specific effects.

Intraoperative positioning may contribute to compressive neuropathy, as discussed by Schwarzman et al^9^ in a large perioperative series. It is more likely, however, that patients in this situation would experience symptoms acutely after surgery rather than months after surgery. Yian et al^10^ reported that rates of compressive neuropathy were not notably different in patients undergoing shoulder arthroplasty compared to a nonsurgical control group. Dua et al^5^ also compared open versus arthroscopic shoulder procedures and found no notable differences in neuropathy rates between the two. Both studies argue that upper-extremity surgery or even upper-extremity surgery type does not fully explain the risk of neuropathy. Our results align with their conclusions; despite procedural differences between THA and TKA, the overall incidence of postoperative CTS and CuTS remained under 1% for each group, with similar distributions of neuropathy type and bilateral and unilateral symptoms. As such, rather than direct anatomic insult to nerves, other factors such as latent susceptibilities unmasked by perioperative stressors or patient-specific characteristics should be considered.

For instance, it is well documented that increasing age is associated with higher incidence of CTS and CuTS, peaking in women between ages 45 and 54 and continuing to rise in men beyond age 60.^11^, ^12^, ^13^ Our findings parallel these trends: the majority of neuropathies in our cohort occurred in older patients, and patients with moderate-to-severe findings on EMG were considerably older than patients who had normal-to-mild EMG results. This age-related susceptibility could reflect age-dependent neural degeneration, diminished tissue resilience, and cumulative subclinical compression. These observations again support the hypothesis that patient-specific factors such as age or preexisting subclinical neuropathy may play a role in postoperative neuropathy risk.

Interestingly, although ACDF patients had the highest observed rate of peripheral neuropathy (3.05%), they were also the youngest cohort to develop a peripheral neuropathy and experienced symptoms considerably sooner. This paradoxical finding suggests a potential double crush syndrome phenomenon, wherein cervical spine pathology can exacerbate distal compressive neuropathies.^14^^,^^15^ The literature is mixed regarding whether cervical spine pathology is a risk factor for worse outcomes after peripheral nerve decompression.^16^, ^17^, ^18^ Our study did not find differences in EMG severity nor postoperative symptom resolution between the ACDF, THA, and TKA cohorts. However, ACDF patients were notably more likely to present with a combined CTS/CuTS diagnoses compared to THA and TKA patients, showing a broader distribution of nerve involvement. Future studies should compare the involved cervical spine levels with the distribution of peripheral neuropathy, investigate associated changes in electrodiagnostic studies, and evaluate differences in patients with cervical radiculopathy versus myelopathy as they are distinct disease processes.

In our study, surgical decompression was associated with a high rate of symptom improvement and conservative management was markedly less effective, particularly for combined neuropathies. These results are consistent with Dua et al,^5^ who found that decompression yielded superior outcomes irrespective of index procedure or EMG severity when compared to nonsurgical treatment. Our data show that 97% of patients with normal-to-mild EMG findings achieved symptom resolution after surgery compared to 88.6% in patients with moderate-to-severe EMG findings. Severity varied by neuropathy type. Specifically, isolated CTS and combined CTS/CuTS were notably more associated with moderate-to-severe findings, whereas isolated CuTS was notably more likely to be graded as mild or normal. This interesting difference may reflect both anatomical and diagnostic factors such as the more constrained anatomy of the carpal tunnel or possibly because of the lower sensitivity of EMG testing for CuTS.^19^ On the contrary, the literature does warn against using EMG severity as a prognostic tool, as studies by Koo et al^20^ and Pohl et al^21^ found little correlation between electrodiagnostic severity and outcomes of CTS and CuTS release. Ultimately, EMG severity may guide patients and surgeons with treatment selection when performed and interpreted accurately, but should not be used as the sole decision making tool.

Median and ulnar neuropathies may be diagnosed following nonupper extremity surgery at low rates comparable to those reported after upper-extremity procedures in the literature. Within this cohort, patients undergoing ACDF demonstrated an earlier time to diagnosis and a broader distribution of nerve involvement, potentially because of double crush phenomenon. Surgical decompression is strongly associated with symptom resolution across neuropathy types. Overall, these findings suggest that distal compressive neuropathies diagnosed after surgeries may often reflect baseline patient susceptibility rather than a direct consequence of the index procedure.

This study is not without limitations. Its retrospective nature introduces inherent selection and information biases and precluded the uniform use of standardized diagnostic tools. Outcome assessment relied primarily on clinical documentation, which introduces subjectivity. EMG data were incomplete, limiting the granularity of subgroup analyses. Additionally, we did not control for patient comorbidities such as diabetes or smoking status which may influence neuropathy risk and treatment outcomes. Treatment selection was nonrandomized and may reflect unmeasured confounders, including symptom duration or severity.

## Conflicts of Interest

No benefits in any form have been received or will be received related directly to this article.
